# Intraventricular hemorrhage clot clearance rate as an outcome predictor in patients with aneurysmal subarachnoid hemorrhage: A retrospective study

**DOI:** 10.1186/s12883-021-02505-0

**Published:** 2021-12-11

**Authors:** Hae Gi Park, Sunghan Kim, Joonho Chung, Chang Ki Jang, Keun Young Park, Jae Whan Lee

**Affiliations:** 1 Department of Neurosurgery, Severance Stroke Center, Severance Hospital, Yonsei University College of Medicine, Seoul, Republic of Korea; 2 Department of Neurosurgery, Bucheon St. Mary’s Hospital, College of Medicine, The Catholic University of Korea, Seoul, Republic of Korea; 3 Department of Neurosurgery, Yongin Severance Hospital, Yonsei University College of Medicine, Yongin, Republic of Korea

**Keywords:** aneurysmal subarachnoid hemorrhage, cerebral intraventricular hemorrhage, patient outcomes assessment, clot clearance rate, modified Graeb score

## Abstract

**Background:**

The development of intraventricular hemorrhage (IVH) in aneurysmal subarachnoid hemorrhage (aSAH) is linked with higher mortality and poor neurological recovery. Previous studies have investigated the effect of the amount and distribution of the initial IVH on the prognosis of aSAH. However, no studies have assessed the relationship between the changes in IVH over time and the prognosis of aSAH. The aim of this study was to analyze the effect of the clearance rate of IVH, which can be represented by the IVH clot clearance rate (CCR), on the outcomes of aSAH.

**Methods:**

The IVH CCR was calculated based on the difference between the initial and follow-up modified Graeb scores (mGS), which were assessed by initial and 7-day follow-up brain computed tomography, respectively. Poor functional outcome was defined as a modified Rankin Scale score of 3-6. Univariate and multivariable analyses were performed to assess the relationships between IVH CCR and other risk factors and the prognosis of patients. Receiver operating characteristic curve analysis was performed to identify cut-off values of IVH CCR for predicting poor functional outcome.

**Results:**

In total, 196 consecutive patients were diagnosed with aSAH between January 2014 and March 2018. According to the inclusion and exclusion criteria, 67 patients were finally included in the study. The univariate analysis revealed that a lower IVH CCR (p<0.001), higher initial mGS (p<0.001), older age (p<0.001), higher initial Hunt and Hess grade (p<0.001), presence of delayed infarction (p=0.03), and presence of shunt-dependent hydrocephalus (p=0.004) were significantly related to poor functional outcome. The multivariable analysis revealed that IVH CCR (odds ratio [OR] 0.941; p=0.029), initial mGS (OR 1.632; p=0.043), age (OR 1.561; p=0.007), initial Hunt and Hess grade (OR 227.296; p=0.030), and delayed infarction (OR 5310.632; p=0.023) were independent predictors of poor functional outcome. Optimal cut-off values of IVH CCR and mGS for poor outcome were 36.27%, and 13.5, respectively (all p< 0.001).

**Conclusions:**

The IVH CCR might have an important predictive value on poor functional outcome in patients with aSAH and IVH, along with initial mGS, age, initial Hunt and Hess grade, and delayed infarction.

## Background

Approximately 30-70% of all cases of aneurysmal subarachnoid hemorrhage (aSAH) are accompanied by intraventricular hemorrhage (IVH), and the development of IVH in aSAH is linked with higher mortality and poor neurological recovery [1–3]. Previous studies have shown that IVH contributes to poor prognosis by causing various complications such as hydrocephalus [1, 3–7], intracranial hypertension [8], fever [9, 10] and ventriculitis [11, 12]. Additionally, IVH can cause shunt-dependent hydrocephalus, which requires permanent cerebrospinal fluid (CSF) diversion [7, 13, 14].

Meanwhile, previous studies have investigated the relationship between the distribution of IVH and prognosis of aSAH, going one step beyond the simple evaluation of aSAH prognosis based on the presence or absence of IVH. Various scoring systems have been suggested for determining the amount of IVH to be used as prognostic values, namely the Fischer grade [15], LeRoux score [16] and Graeb score [17]. In particular, Czorlich et al. demonstrated that the Graeb score seems to be superior to the Fisher and Le Roux scores in predicting mortality in patients with aSAH [18]. The modified Graeb score (mGS) was subsequently developed for more detailed scoring of IVH in patients with IVH [19]. mGS was found to be superior to the original Graeb score in terms of IVH evaluation in predicting the clinical course and outcome of patients with aSAH [20].

Prior studies have suggested that initial IVH volume or density can cause poor outcomes and fatality in patients with aSAH [4, 7, 21]. However, no studies have assessed the relationship between the changes in IVH over time and the prognosis of aSAH. Since previous studies have shown that the presence and amount of IVH affects the prognosis of aSAH, we hypothesized that the IVH clot clearance rate (CCR) will also affect the clinical outcome of aSAH with IVH. In this study, IVH CCR, which indicates the change in IVH with time, was calculated using the sequential change in the amount of mGS. We aimed to evaluate the IVH CCR as an outcome predictor for patients with aSAH and IVH by retrospectively analyzing the functional outcome at 3-month follow-up. Furthermore, outcome predictors that affect the prognosis of aSAH with IVH, other than IVH CCR, were also investigated.

## Methods

### Patient selection

**Fig. 1:**
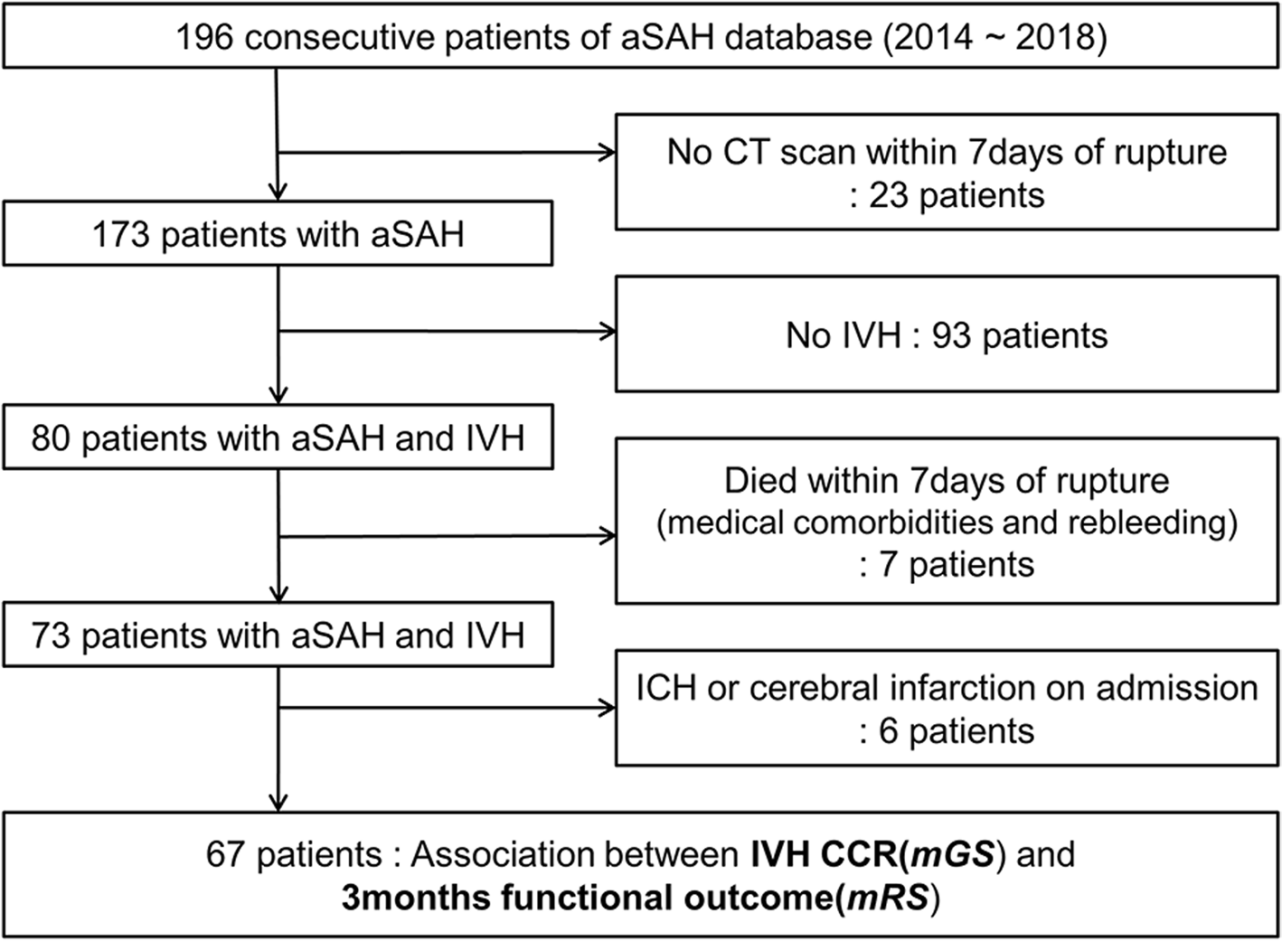
Description of the patient cohort used in this study. *IVH* intraventricular hemorrhage; *CCR* clot clearance rate; *aSAH* aneurysmal subarachnoid hemorrhage; *CT* computed tomography; *ICH* intracerebral hemorrhage; *mGS* modified Graeb Scale; *mRS* modified Rankins Scale

We retrospectively reviewed prospectively collected data regarding intracerebral aneurysms from a single institution’s database. In total, 196 consecutive patients were diagnosed with aSAH between January 2014 and March 2018 (Fig. 1). The inclusion criteria of this study were as follows: (1) SAH was caused by the rupture of intracerebral aneurysm; (2) aSAH was accompanied by IVH at the time of diagnosis; (3) the ruptured aneurysm was treated either by microsurgery or by an endovascular method; (4) follow-up brain computed tomography (CT) was performed within 7 days to assess the IVH CCR and; (5) modified Rankins Scale (mRS) score at 3 months after discharge was identifiable via medical records. The exclusion criteria were as follows: (1) patients with any history of stroke that may affect the prognosis of aSAH other than IVH, such as a history of treatment for a ruptured or unruptured aneurysm, or a history of ischemic stroke; (2) patients presenting with any stroke-related lesion that may affect the prognosis of aSAH other than IVH, such as intracerebral hemorrhage, cerebral infarction, or vascular malformation; (3) patients who died within 7 days of admission, and; (4) patients with clinical deterioration due to rebleeding of the aneurysm. Intraventricular hemorrhage was confirmed in 80 of 173 patients, and 23 patients did not undergo follow-up CT within 7 days of aneurysmal rupture. After excluding 13 patients according to the exclusion criteria, 67 patients were finally included in the study.

Demographic, clinical, and radiographic data of the patients were collected based on electrical medical records, and data obtained from the aneurysm database were reviewed and compared for each group. The clinical condition on admission was graded according to the Hunt and Hess (H-H) grading system and radiologic condition on admission was assessed by SAH thickness on initial CT. The main outcome variable was the mRS score at 3-month follow-up, which was dichotomized between mRS scores of 0–2 and 3–6. The included patients were divided into two groups according to the mRS score at 3-month follow up (good outcome group, mRS score of 0–2 vs. poor outcome group, mRS score of 3–6). Delayed ischemic neurologic deficit (DIND), delayed infarction, and shunt-dependent hydrocephalus were investigated along with IVH CCR as prognostic factors for aSAH. Delayed ischemic neurologic deficit refers to symptomatic vasospasm that requires treatment identified by angiography, based on Kramer's criteria [1]. Delayed infarction was diagnosed if permanent cerebral infarction caused by DIND was confirmed by imaging. Shunt-dependent hydrocephalus was determined by clinical and radiologic signs of hydrocephalus which requires permanent CSF diversion within the follow-up period. This retrospective study was approved by the institutional review board of Severance hospital at Yonsei University College of Medicine (subject number: 4-2021-0828) and Bucheon St. Mary's hospital at the Catholic University of Korea (subject number: HC21RASI0072), and the requirement for informed consent was waived.

### Treatment strategies for aneurysmal subarachnoid hemorrhage

All aSAH cases included in this study were treated according to the following protocol: (1) ruptured cerebral aneurysms were repaired within 48 hours by microsurgery or endovascular method; (2) the treatment plan was determined by multidisciplinary discussions among experienced neurosurgeons, neurointerventionists, and radiologists; (3) optimal medical treatment for preventing post-aSAH complications such as cerebral edema, vasospasm, and hydrocephalus were performed including close monitoring in intensive care units, administration of Nimodipine, and regular follow-up via transcranial doppler ultrasonography. No antiplatelet or anticoagulation medication was used to prevent delayed ischemic event; (4) in case of acute hydrocephalus with clinical deterioration despite treatment, extraventricular drainage (EVD) was considered; (5) the EVD was maintained for up to two weeks, and if the EVD was intended to be maintained for more than two weeks, a change of EVD or permanent CSF diversion was considered contextually and; (6) the injection of intraventricular tissue plasminogen activator for the purpose of clot lysis was not considered.

### Assessment of the clot clearance rate of intraventricular hemorrhage

The mGS was calculated by the method introduced by Morgan et al. [19]. The mGS incorporates IVH scores in relation to its anatomical extension. The maximum score of 32 indicates that every compartment is filled with blood and expanded. A score of 0 indicates no IVH. To evaluate the IVH CCR, initial and follow-up mGS was assessed by initial and 7-day follow-up brain CT, respectively. The IVH CCR was calculated as follows:

$$\mathrm{IVH}\ \mathrm{CCR}\ \left(\%\right):\left[\left(\mathrm{initial}\ \mathrm{mGS}-\mathrm{follow}\ \mathrm{up}\ \mathrm{mGS}\right)/\mathrm{initial}\ \mathrm{mGS}\right]\ \mathrm{x}\ 100$$

All mGS values were measured by two independent investigators. If the measurements of the two investigators differed considerably, another neuroradiologist reviewed the data and a final consensus was reached by the three investigators regarding the results.

## Statistical analysis

The Student’s t-test was used for analyses of continuous variables and Pearson χ2 test or Fisher’s exact test was used for the analyses of categorical variables. Continuous variables were described as mean ± standard deviation (SD) and categorical variables were summarized as frequencies and percentages (%). A univariate logistic regression analysis was performed to determine the association of poor outcome with factors and characteristics associated to aSAH with IVH. A multivariate logistic regression analysis was performed for variables with an unadjusted effect, and p values <0.05 in the univariate analysis was used to determine independent associations between poor outcome and characteristics of aSAH with IVH. The threshold value for the cut-off point of the continuous variable among the outcome predictors for aSAH with IVH was determined using the receiver operating characteristics (ROC) analysis, and the diagnostic performance of the each variable was assessed. Pearson’s rank correlation coefficient was used to extrapolate the relationships between the continuous variable among the outcome predictors and IVH CCR. All p values <0.05 were considered statistically significant. All statistical analyses were performed using SPSS Statistics 23.0 (IBM Corp., Amonk, NY, USA).

## Results

### Outcome predictors for aneurysmal subarachnoid hemorrhage with intraventricular hemorrhage

Among the 67 patients, poor outcome was observed in 29 (43.3%) patients. A comparison of the good outcome group and the poor outcome group indicated no significant differences in baseline characteristics of the patients besides age (Table 1). The univariate analysis revealed that old age (p<0.001), higher initial H-H grade (p<0.001), presence of delayed infarction (p=0.03), and presence of shunt-dependent hydrocephalus (p=0.004) were significantly associated with poor functional outcome. However, sex, SAH thickness, location of aneurysm, and DIND were not significantly related to poor functional outcome. The initial mGS of the poor outcome group was higher than that of the good outcome group (16.8± 5.7 vs. 10.6± 5.4, p<0.001). The IVH CCR in the poor outcome group was lower than that in the good outcome group (28.3± 24.0 vs. 67.4± 29.1, p<0.001). The multivariable analysis revealed that old age (odds ratio [OR], 1.561; 95% confidence interval [CI], 1.126–2.162; p=0.007), higher initial H-H grade (OR, 227.296; 95% CI 1.715–30119.199; p=0.030), and presence of delayed infarct (OR, 5310.632; 95% CI 3.209–8788298.374; p=0.023) were independent predictors of poor outcome. Additionally, a higher initial mGS (OR, 1.632; 95% CI, 1.015–2.622; p=0.043) and low rate of IVH CCR (OR, 0.941; 95% CI, 0.890–0.994; p=0.029) were independent predictors of unfavorable functional outcome. These results are summarized in Table 2.

**Table 1:** Comparison of baseline characteristics between two groups

Variables	Good outcome (n=38)	Poor outcome (n=29)	p value
Age (years)	55.9 ± 11.3	74.5 ± 8.9	<.0001
Female	33 (86.8)	22 (75.9)	0.338
Risk factors			
Hypertension	14 (36.8)	8 (27.6)	0.447
Diabetes mellitus	9 (23.7)	10 (34.5)	0.415
Dyslipidemia	8 (21.1)	5 (17.2)	0.764
Coronary artery disease	1 (2.6)	3 (10.3)	0.308*
Smoking	3 (7.9)	4 (13.8)	0.456*
Location of aneurysm			0.338
Anterior circulation	33 (86.8)	22 (75.9)	
Posterior circulation	5 (13.2)	7 (24.1)	
Treatment method			0.271
Micosurgery	8 (21.1)	10 (34.5)	
Endovascular method	30 (78.9)	19 (65.5)	
Extraventricular drainage	16 (42.1)	11 (37.9)	0.804

Values are presented as mean ± standard deviation or number (%).

*p value using Fisher’s exact test.

**Table 2:** Univariate and multivariable analysis of risk factors for poor functional outcomes after aneurysmal subarachnoid hemorrhage with intraventricular hemorrhage

			Univariate analysis	Multivariable analysis	
Parameter	Good outcome (n=38)	Poor outcome (n=29)	P-value	OR (95% CI)	P-value
Age (years)	55.9 ± 11.3	74.5 ± 8.9	<0.001	1.561 (1.126–2.162)	0.007
Female	33 (86.8)	22 (75.9)	0.200		
Initial H-H grade			<0.001	227.296 (1.715–30119.199)	0.030
1-3	33 (86.8)	6 (20.7)			
4-5	5 (13.2)	23 (79.3)			
SAH thickness			0.173		
Thin (<1mm)	16 (42.1)	12 (41.4)			
Thick (>1mm)	22 (57.9)	17 (58.6)			
Location of aneurysm			0.200		
Anterior circulation	33 (86.8)	22 (75.9)			
Posterior circulation	5 (13.2)	7 (24.1)			
DIND	3 (7.9)	3 (10.3)	0.526		
Delayed infarction	2 (5.3)	7 (24.1)	0.030	5310.632 (3.209–8788298.374)	0.023
Shunt-dependent hydrocephalus	6 (15.8)	14 (48.3)	0.004	0.025 (0–4.957)	0.171
Initial mGS	10.6 ± 5.4	16.8 ± 5.7	<0.001	1.632 (1.015–2.622)	0.043
IVH CCR (%)	67.4 ± 29.1	28.3 ± 24.0	<0.001	0.941 (0.890–0.994)	0.029

Values are presented as mean ± standard deviation or number (%).

*OR* odds ratio; *CI* confidence interval; *H-H* Hunt & Hess; *SAH* subarachnoid hemorrhage; *CHC* chronic hydrocephalus; *DIND* delayed ischemic neurologic deficiet; *mGS* modified Graeb score; *IVH* intraventricular hemorrhage; *CCR* intraventricular clot clearance rate

### Associations of outcome predictors and functional outcomes of aneurysmal subarachnoid hemorrhage with intraventricular hemorrhage

**Fig. 2:**
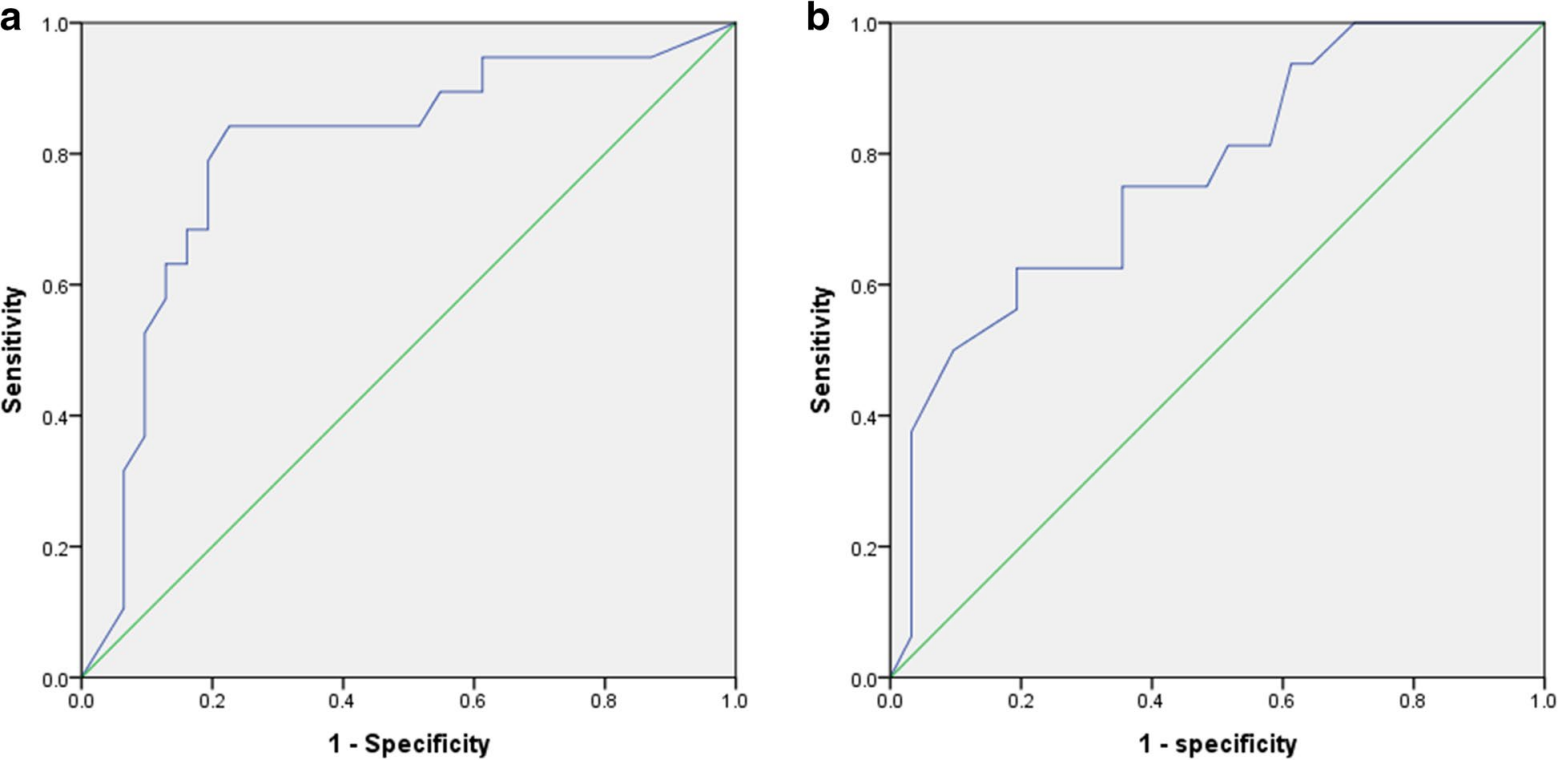
The ROC curve of the IVH CCR and the initial mGS for the outcome prediction. (a) The cut-off value of the IVH CCR was 36.27% (AUC 0.838, P< 0.001). (b) The cut-off value of the initial mGS was 13.5 (AUC 0.787, P < 0.001). *ROC* receiver operating characteristic; *IVH* intraventricular hemorrhage; *CCR* intraventricular clot clearance rate; *mGS* modified Graeb score; *AUC* area under the curve

**Fig. 3:**
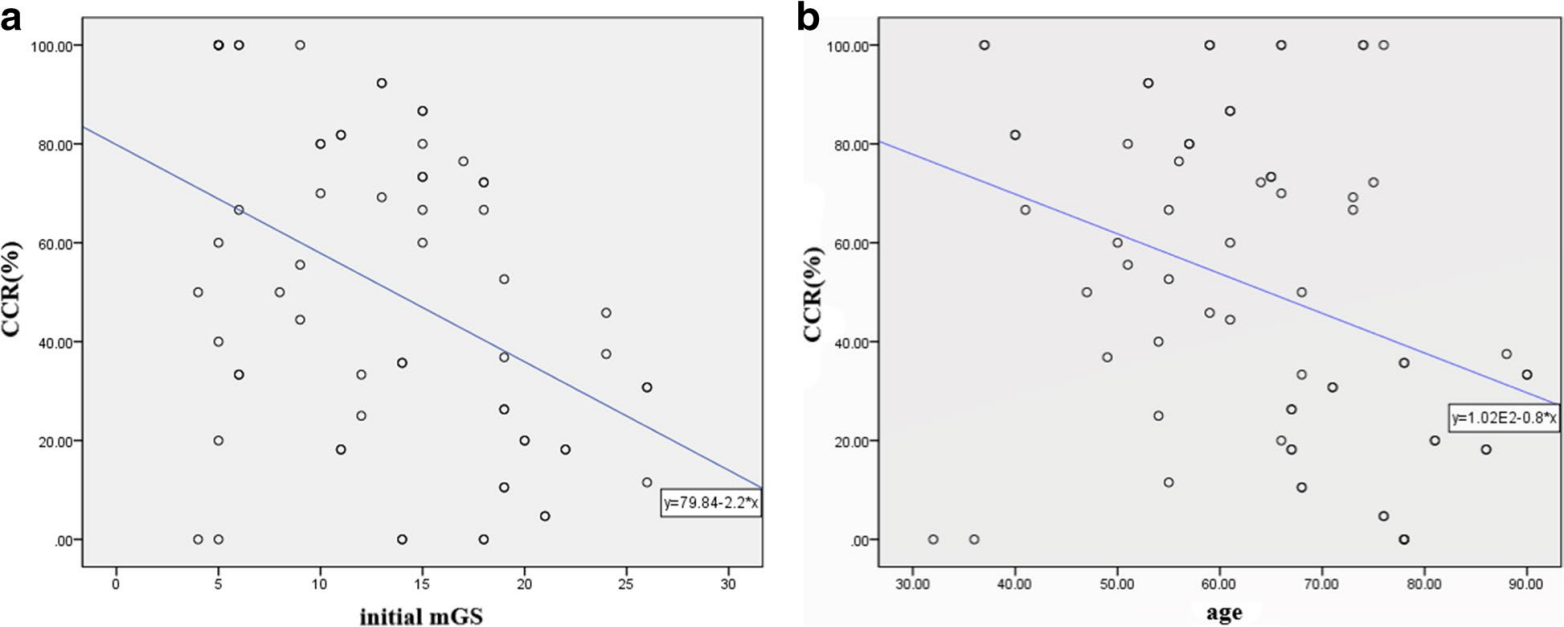
Correlation of (a) initial mGS, (b) age, and IVH CCR. A negative correlation was noted for both initial mGS (r=-0.422, p<0.001) and age (r=-0.336, p<0.001) with IVH CCR. *mGS* modified Graeb score; *IVH* intraventricular hemorrhage; *CCR* intraventricular clot clearance rate

The ROC curve for the cut-off value of the IVH CCR showed an area under the curve (AUC) of 0.838 (Fig. 2a). The cut-off value of the IVH CCR was determined to be 36.27%, for which the sensitivity and specificity were 84.2% and 75.9% respectively. The ROC curve for the initial mGS for the prediction of poor outcome in aSAH with IVH showed an AUC of 0.787 (Fig. 2b). The cut-off value of the initial mGS was determined to be 13.5, for which the sensitivity and specificity were 75.9% and 68.4% respectively. Pearson’s rank correlation coefficient was used to extrapolate the relationships between initial mGS (Fig. 3a), age (Fig. 3b), and IVH CCR. The correlation test revealed a negative correlation for both initial mGS (r=-0.422, p<0.001) and age (r=-0.336, p<0.001) with IVH CCR.

## Discussion

In this observational cohort of 67 patients with aSAH and IVH, it was found that a low rate of IVH CCR independently predicted unfavorable functional outcome in patients with aSAH and IVH along with old age, higher initial H-H grade, presence of delayed infarction, and higher initial mGS. Furthermore, initial mGS was found to be negatively correlated with IVH CCR. These results suggest that not only initial IVH volume or density but also IVH CCR is related to the functional outcome in aSAH with IVH.

Previous studies have reported the impact of initial IVH burden on early complications and fatality in cases of aSAH. Czorlich et al .[18] retrospectively analyzed aSAH in 206 consecutive patients and discussed a significant association between initial IVH severity and case fatality rate. Moreover, Darkwah Oppong et al .[4] reported the result of a retrospective study of aSAH with IVH in 487 patients that severity of IVH was related to acute hydrocephalus and poor outcome at 5-month follow-up, and that the outcome was closely related to early complications of aSAH such as cerebral infarction, early mortality, and primary craniectomy. Eagles et al .[22] observed that delayed cerebral ischemia in aSAH can be predicted by initial IVH volume calculated using mGS. As IVH adversely affects the prognosis of aSAH in various ways, it can be hypothesized that if IVH clearance is delayed, these adverse effects will be maintained for a long time, resulting in a poor prognosis. This hypothesis is corroborated by the current findings that a low rate of IVH CCR is an independent prognostic factor of poor functional outcome in aSAH with IVH.

Hemorrhaging that extends to the ventricular system damages the brain via several possible mechanisms. The severity of hemorrhaging has a direct mass effect on the adjacent neural tissue, which can cause neural damage and cerebral edema [23]. Moreover, hemorrhage in the ventricular system blocks the circulatory balance of the CSF, and it can negatively affect the regulation of intracranial pressure and cerebral perfusion [24]. IVH also results in an increased inflammatory reaction in the CSF [25]. Moreover, the greater is the initial IVH burden, the greater is the CSF inflammatory reaction. Therefore, a slower IVH CCR might result in additional exposure with respect to the CSF inflammatory reaction. This might be the one of reasons why the IVH CCR in the early hemorrhage period is related to poor functional outcome, as shown by our results.

There were several limitations to this study. First, it was designed retrospectively and included a small number of patients from a single center. Second, even though two observers assessed the lesions independently, bias could have affected the results. Third, the efficacy of aneurysm treatments performed, such as clips and coils, is expected to affect the patient's prognosis; however, this was not considered in this study. Finally, the effect of CSF drainage on IVH CCR and patient outcomes was not evaluated. Therefore, further prospective studies with well-adjusted confounding variables are needed to better understand the relationship between IVH CCR and functional outcomes of aSAH with IVH.

## Conclusions

The results of this study suggest that the IVH CCR might have an important predictive value on poor functional outcome in patients with aSAH and IVH, along with age, H-H grade, delayed infarct, and initial mGS. Additional studies with larger sample sizes are needed to provide more evidence in this regard.

## Data Availability

The relevant anonymized patient level data are available upon reasonable request from the corresponding author, Jae Whan Lee.

## Abbreviations

aSAH: aneurysmal subarachnoid hemorrhage
AUC: area under the curve
CCR: clot clearance rate
CI: confidence interval
CSF: cerebrospinal fluid
CT: computed tomography
DIND: Delayed ischemic neurologic deficit
EVD: extraventricular drainage
H-H: Hunt and Hess
IVH: intraventricular hemorrhage
mGS: modified Graeb score
mRS: modified Rankins Scale
OR: odds ratio
ROC: receiver operating characteristics
SD: standard deviation
